# Crystal structure of di­cyclo­hexyl­ammonium nitrate(V)

**DOI:** 10.1107/S2056989015019386

**Published:** 2015-10-24

**Authors:** Tomasz Rojek, Ewa Matczak-Jon

**Affiliations:** a Department of Chemistry, Wrocław University of Technology, 27 Wybrzeże Wyspiańskiego St., 50-370 Wrocław, Poland

**Keywords:** crystal structure, di­cyclo­hexyl­ammonium salts, nitrate(V) salts, hydrogen bonding

## Abstract

In the title mol­ecular salt, C_12_H_24_N^+^·NO_3_^−^, the cyclohexyl rings adopt chair conformations with the exocyclic C—N bonds in equatorial orientations. In the crystal, a bifurcated N—H⋯(O,O) hydrogen bond links the cation to the anion; the ion pairs are linked *via* C—H⋯O hydrogen bonds, forming layers in the *ac* plane.

## Related literature   

For the crystal structure of di­cyclo­hexyl­ammonium nitrate(III), see: Golobič *et al.* (1999 ▸). For other crystal structures of di­cyclo­hexyl­ammonium salts, see: Ng (1995 ▸); Bi *et al.* (2002 ▸); Lo & Ng (2008 ▸); Khawar Rauf *et al.* (2008 ▸); Selvakumaran *et al.* (2011 ▸); Ndoye *et al.* (2014 ▸). For crystal structures of carboxyl­ate salts with the di­cyclo­hexyl­ammonium cation belonging to the low mol­ecular weight gelators (LMWGs) class of compounds and exhibiting gelling properties, see: Trivedi *et al.* (2004 ▸, 2005 ▸); Sahoo & Dastidar (2012 ▸); Rojek *et al.* (2015 ▸).
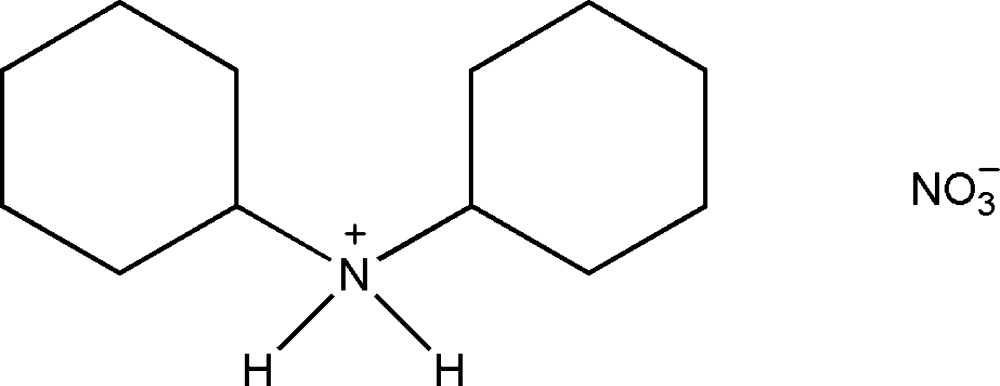


## Experimental   

### Crystal data  

C_12_H_24_N^+^·NO_3_^−^
*M* *_r_* = 244.33
Orthorhombic, 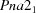

*a* = 8.436 (2) Å
*b* = 18.682 (5) Å
*c* = 8.427 (3) Å
*V* = 1328.1 (7) Å^3^
*Z* = 4
Mo *K*α radiation
μ = 0.09 mm^−1^
*T* = 100 K
0.45 × 0.41 × 0.36 mm

### Data collection  

Kuma KM-4 difractometer with a CCD camera diffractometer
Absorption correction: multi-scan (*CrysAlis RED*; Oxford Diffraction, 2010 ▸) *T*_min_ = 0.962, *T*_max_ = 0.969
9001 measured reflections
1759 independent reflections
1699 reflections with *I* > 2σ(*I*)
*R*_int_ = 0.035

### Refinement  

*R*[*F* ^2^ > 2σ(*F* ^2^)] = 0.035
*wR*(*F* ^2^) = 0.089
*S* = 1.09
1759 reflections
162 parameters
1 restraint
H atoms treated by a mixture of independent and constrained refinement
Δρ_max_ = 0.21 e Å^−3^
Δρ_min_ = −0.19 e Å^−3^

### 

Data collection: *CrysAlis PRO* (Agilent, 2011 ▸); cell refinement: *CrysAlis PRO*; data reduction: *CrysAlis PRO*; program(s) used to solve structure: *SHELXS97* (Sheldrick, 2008 ▸); program(s) used to refine structure: *SHELXL97* (Sheldrick, 2008 ▸); molecular graphics: *DIAMOND* (Brandenburg, 1999 ▸); software used to prepare material for publication: *publCIF* (Westrip, 2010 ▸).

## Supplementary Material

Crystal structure: contains datablock(s) I, New_Global_Publ_Block. DOI: 10.1107/S2056989015019386/su5222sup1.cif

Structure factors: contains datablock(s) I. DOI: 10.1107/S2056989015019386/su5222Isup2.hkl

Supporting information file. DOI: 10.1107/S2056989015019386/su5222Isup3.txt

Supporting information file. DOI: 10.1107/S2056989015019386/su5222Isup4.cml

**Fig. 1.**
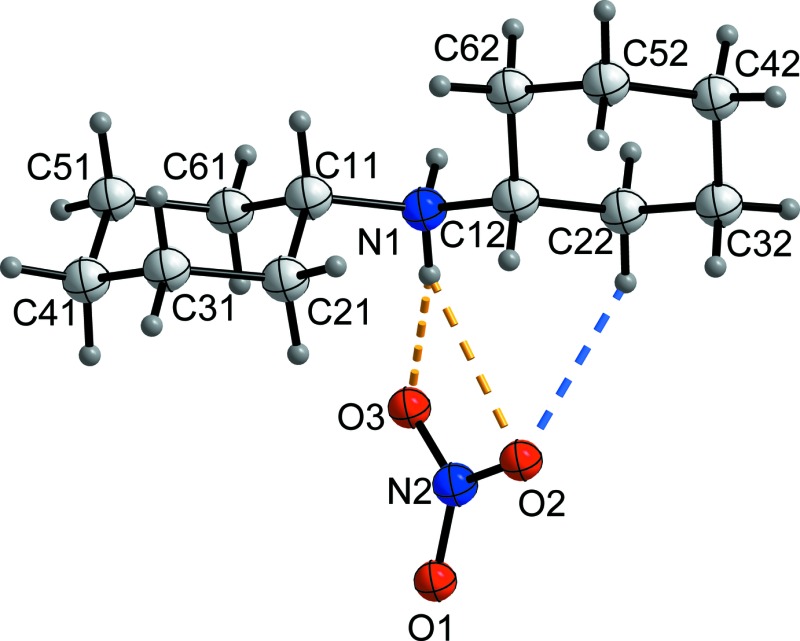
The asymmetric unit of the title molecular salt, showing the atom-numbering scheme and the symmetry-independent hydrogen bonds (orange and light-blue dashed lines; see Table 1). Displacement ellipsoids are drawn at the 50% probability level.

**Fig. 2.**
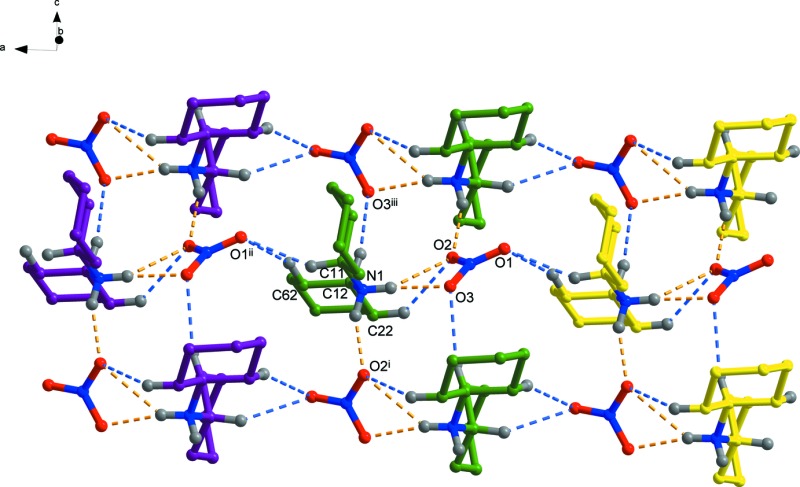
A view along the b axis of the crystal packing of the title molecular salt, showing the hydrogen-bonded chains assembled into a layer in the ac plane. Hydrogen bonds are drawn as yellow and light-blue dashed lines (see Table 1). H atoms on C atoms of the cyclohexane rings not involved in hydrogen bonds have been omitted for clarity.

. DOI: 10.1107/S2056989015019386/su5222fig1.tif
The asymmetric unit of the title mol­ecular salt, showing the atom-numbering scheme and the symmetry-independent hydrogen bonds (orange and light-blue dashed lines; see Table 1). Displacement ellipsoids are drawn at the 50% probability level.

b ac . DOI: 10.1107/S2056989015019386/su5222fig2.tif
A view along the *b* axis of the crystal packing of the title mol­ecular salt, showing the hydrogen-bonded chains assembled into a layer in the *ac* plane. Hydrogen bonds are drawn as yellow and light-blue dashed lines (see Table 1). H atoms on C atoms of the cyclo­hexane rings not involved in hydrogen bonds have been omitted for clarity.

CCDC reference: 1431025

Additional supporting information:  crystallographic information; 3D view; checkCIF report

## Figures and Tables

**Table 1:** Hydrogen-bond geometry (, )

*D*H*A*	*D*H	H*A*	*D* *A*	*D*H*A*
N1H1*N*O2	0.91(2)	2.56(2)	3.292(2)	138.2(17)
N1H1*N*O3	0.91(2)	2.01(2)	2.8988(19)	166.7(19)
N1H2*N*O2^i^	0.86(2)	1.98(2)	2.799(2)	157.6(19)
C11H11O1^ii^	1.00	2.45	3.347(2)	149
C12H12O3^iii^	1.00	2.52	3.456(3)	156
C22H22*B*O2	0.99	2.53	3.309(2)	136
C62H62*A*O1^ii^	0.99	2.59	3.506(2)	153

Symmetry codes: (i) 
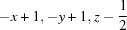
; (ii) 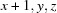; (iii) 
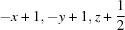
.

## supplementary crystallographic information

### S1. Comment

The di­cyclo­hexyl­ammonium cation has been widely used in the preparation of crystalline salts like chloride (Ng, 1995), nitrate(III) (Golobič *et al.*, 1999), tungstate (Bi *et al.*, 2002), bromide (Lo & Ng, 2008), thio­cyanate (Khawar Rauf *et al.*, 2008; Selvakumaran *et al.*, 2011) or sulfate­(VI) (Ndoye *et al.*, 2014) salts. In recent years, there has been increased inter­est in di­cyclo­hexyl­ammonium carboxyl­ate salts due to their potential applications as materials capable of immobilizing organic solvents to form gels, known as low molecular weight gelators - LMWGs (Trivedi *et al.*, 2004, 2005; Sahoo & Dastidar, 2012; Rojek *et al.*, 2015).

The title molecular salt, Fig. 1, consists of an ion pair comprising a di­cyclo­hexyl­ammonium cation connected to a nitrate(V) anion by N1—H1N···O3 and N1—H1N···O2 hydrogen bonds (Table 1). Additionally, the ion pair is stabilized by a C22—H22B···O2 inter­action (Fig. 1 and Table 1). The N2—O2 and N2—O3 bond lengths of the nitrate(V) anion are almost equal [1.258 (2) and 1.255 (2) Å, respectively] and longer than the N2—O1 bond length [1.2353 (18) Å]. The C11—N1—C12 angle in the di­cyclo­hexyl­ammonium cation [117.34 (12)°] is larger than expected for a tetra­hedral N atom. This is attributed to the steric hindrance imposed by the cyclo­hexane rings, each of which adopt a chair conformation. The N1—C11 and N1—C12 bond lengths [1.5077 (19) and 1.510 (2) Å, respectively] are similar to those observed for other di­cyclo­hexyl­ammonium salts (Golobič *et al.*, 1999).

In the crystal, the N1—H2N···O2^i^ hydrogen bonds combine ion pairs into infinite chains parallel to the *c* axis. The chains are additionally stabilized by C12—H12···O3^iii^ contacts and further packed in a parallel fashion by means of C62—H62A···O1^ii^ and C11—H11···O1^ii^ (symmetry codes as in Table 1) inter­actions giving rise to layers in the *ac* plane (Fig. 2).

### S2. Synthesis and crystallization

Di­cyclo­hexyl­amine (1 mmol, 201 ml) was added to methanol (4 ml) under vigorous stirring. The clear solution was combined with nitric(V) acid (1 M, 1 ml) and stirred for 20 min. The resulting solution was left to crystallize at room temperature. After one week, large block-shaped colourless single crystals of the title salt suitable for X-ray diffraction analysis were obtained.

### S3. Refinement

The N-bound H atoms were located in a difference Fourier map and freely refined. The C-bound H atoms were positioned geometrically and refined using a riding model; C—H = 0.99 Å with U_iso_(H) = 1.2U_eq_(C).

### Crystal data

**Table:**

C_12_H_24_N^+^·NO_3_^−^	*F*(000) = 536
*M**_r_* = 244.33	*D*_x_ = 1.222 Mg m^−^^3^
Orthorhombic, *P**n**a*2_1_	Mo *K*α radiation, λ = 0.71073 Å
Hall symbol: P 2c -2n	Cell parameters from 6847 reflections
*a* = 8.436 (2) Å	θ = 3–29°
*b* = 18.682 (5) Å	µ = 0.09 mm^−^^1^
*c* = 8.427 (3) Å	*T* = 100 K
*V* = 1328.1 (7) Å^3^	Block, colorless
*Z* = 4	0.45 × 0.41 × 0.36 mm

### Data collection

**Table:**

Kuma KM-4 difractometer with a CCD camera diffractometer	1699 reflections with *I* > 2σ(*I*)
Radiation source: normal focus sealed tube	*R*_int_ = 0.035
ω scans	θ_max_ = 28.7°, θ_min_ = 3.3°
Absorption correction: multi-scan (*CrysAlis RED*; Oxford Diffraction, 2010)	*h* = −9→11
*T*_min_ = 0.962, *T*_max_ = 0.969	*k* = −24→22
9001 measured reflections	*l* = −11→11
1759 independent reflections	

### Refinement

**Table:**

Refinement on *F*^2^	Primary atom site location: structure-invariant direct methods
Least-squares matrix: full	Secondary atom site location: difference Fourier map
*R*[*F*^2^ > 2σ(*F*^2^)] = 0.035	Hydrogen site location: inferred from neighbouring sites
*wR*(*F*^2^) = 0.089	H atoms treated by a mixture of independent and constrained refinement
*S* = 1.09	*w* = 1/[σ^2^(*F*_o_^2^) + (0.051*P*)^2^ + 0.1971*P*] where *P* = (*F*_o_^2^ + 2*F*_c_^2^)/3
1759 reflections	(Δ/σ)_max_ < 0.001
162 parameters	Δρ_max_ = 0.21 e Å^−^^3^
1 restraint	Δρ_min_ = −0.19 e Å^−^^3^

### Special details

**Table:**

Geometry. All e.s.d.'s (except the e.s.d. in the dihedral angle between two l.s. planes) are estimated using the full covariance matrix. The cell e.s.d.'s are taken into account individually in the estimation of e.s.d.'s in distances, angles and torsion angles; correlations between e.s.d.'s in cell parameters are only used when they are defined by crystal symmetry. An approximate (isotropic) treatment of cell e.s.d.'s is used for estimating e.s.d.'s involving l.s. planes.
Refinement. Refinement of *F*^2^ against ALL reflections. The weighted *R*-factor *wR* and goodness of fit *S* are based on *F*^2^, conventional *R*-factors *R* are based on *F*, with *F* set to zero for negative *F*^2^. The threshold expression of *F*^2^ > σ(*F*^2^) is used only for calculating *R*-factors(gt) *etc*. and is not relevant to the choice of reflections for refinement. *R*-factors based on *F*^2^ are statistically about twice as large as those based on *F*, and *R*- factors based on ALL data will be even larger.

### Fractional atomic coordinates and isotropic or equivalent isotropic displacement parameters (Å^2^)

**Table:**

	*x*	*y*	*z*	*U*_iso_*/*U*_eq_	
N1	0.67955 (15)	0.52358 (7)	0.34329 (17)	0.0172 (3)	
H1N	0.575 (3)	0.5334 (10)	0.348 (3)	0.022 (5)*	
H2N	0.703 (2)	0.5150 (11)	0.245 (3)	0.020 (5)*	
C11	0.76844 (18)	0.58933 (8)	0.39662 (19)	0.0174 (3)	
H11	0.8844	0.5812	0.3802	0.021*	
C21	0.7391 (2)	0.60372 (9)	0.5718 (2)	0.0209 (3)	
H21A	0.7773	0.5626	0.6354	0.025*	
H21B	0.6239	0.6091	0.5909	0.025*	
C31	0.8252 (2)	0.67195 (8)	0.6241 (2)	0.0236 (3)	
H31A	0.7994	0.6822	0.7365	0.028*	
H31B	0.9411	0.6644	0.6163	0.028*	
C41	0.7782 (2)	0.73593 (8)	0.5221 (2)	0.0249 (3)	
H41A	0.8415	0.7782	0.5537	0.030*	
H41B	0.6649	0.7472	0.5400	0.030*	
C51	0.8053 (2)	0.72023 (9)	0.3456 (2)	0.0256 (3)	
H51A	0.7681	0.7613	0.2815	0.031*	
H51B	0.9202	0.7140	0.3256	0.031*	
C61	0.71676 (19)	0.65265 (8)	0.2950 (2)	0.0213 (3)	
H61A	0.6012	0.6602	0.3068	0.026*	
H61B	0.7390	0.6424	0.1820	0.026*	
C12	0.71252 (17)	0.45456 (8)	0.4304 (2)	0.0173 (3)	
H12	0.6777	0.4601	0.5432	0.021*	
C22	0.61395 (18)	0.39582 (8)	0.3534 (2)	0.0202 (3)	
H22A	0.6447	0.3906	0.2406	0.024*	
H22B	0.5003	0.4089	0.3575	0.024*	
C32	0.64019 (18)	0.32501 (8)	0.4401 (2)	0.0224 (3)	
H32A	0.6010	0.3291	0.5504	0.027*	
H32B	0.5791	0.2867	0.3866	0.027*	
C42	0.81560 (18)	0.30512 (8)	0.4419 (2)	0.0225 (3)	
H42A	0.8304	0.2606	0.5040	0.027*	
H42B	0.8517	0.2958	0.3320	0.027*	
C52	0.91573 (19)	0.36473 (8)	0.5142 (2)	0.0231 (3)	
H52A	1.0292	0.3516	0.5073	0.028*	
H52B	0.8882	0.3701	0.6278	0.028*	
C62	0.88884 (18)	0.43628 (8)	0.4286 (2)	0.0206 (3)	
H62A	0.9496	0.4746	0.4822	0.025*	
H62B	0.9266	0.4327	0.3176	0.025*	
N2	0.27878 (15)	0.55034 (7)	0.44418 (19)	0.0209 (3)	
O1	0.15367 (14)	0.57836 (7)	0.48831 (17)	0.0325 (3)	
O2	0.33397 (19)	0.49638 (8)	0.51433 (18)	0.0398 (4)	
O3	0.35579 (15)	0.57519 (7)	0.32934 (17)	0.0318 (3)	

### Atomic displacement parameters (Å^2^)

**Table:**

	*U*^11^	*U*^22^	*U*^33^	*U*^12^	*U*^13^	*U*^23^
N1	0.0173 (6)	0.0172 (6)	0.0172 (7)	0.0010 (5)	−0.0018 (5)	−0.0027 (5)
C11	0.0183 (7)	0.0165 (7)	0.0176 (7)	0.0002 (5)	−0.0001 (6)	−0.0019 (5)
C21	0.0260 (7)	0.0202 (7)	0.0165 (7)	−0.0022 (6)	−0.0009 (6)	−0.0014 (6)
C31	0.0288 (8)	0.0199 (7)	0.0220 (8)	0.0000 (6)	−0.0035 (6)	−0.0047 (6)
C41	0.0274 (8)	0.0179 (7)	0.0293 (8)	0.0012 (6)	−0.0015 (7)	−0.0026 (7)
C51	0.0314 (8)	0.0209 (7)	0.0245 (8)	−0.0029 (6)	0.0017 (7)	0.0025 (7)
C61	0.0256 (8)	0.0199 (7)	0.0184 (7)	0.0001 (6)	−0.0003 (6)	0.0021 (6)
C12	0.0170 (6)	0.0162 (6)	0.0187 (7)	0.0009 (5)	−0.0004 (6)	−0.0003 (6)
C22	0.0173 (7)	0.0189 (7)	0.0242 (8)	−0.0007 (5)	−0.0013 (6)	−0.0025 (6)
C32	0.0225 (7)	0.0187 (7)	0.0261 (8)	−0.0011 (5)	0.0034 (7)	−0.0007 (6)
C42	0.0236 (7)	0.0188 (7)	0.0252 (8)	0.0017 (6)	0.0029 (7)	−0.0004 (7)
C52	0.0212 (7)	0.0227 (7)	0.0253 (7)	0.0033 (6)	−0.0045 (6)	0.0005 (7)
C62	0.0178 (7)	0.0196 (7)	0.0243 (8)	0.0002 (5)	−0.0031 (6)	−0.0009 (6)
N2	0.0202 (6)	0.0229 (6)	0.0197 (6)	−0.0009 (5)	−0.0012 (5)	−0.0013 (5)
O1	0.0197 (6)	0.0370 (7)	0.0409 (8)	0.0041 (5)	0.0034 (5)	−0.0079 (6)
O2	0.0594 (9)	0.0360 (7)	0.0239 (6)	0.0236 (6)	0.0088 (7)	0.0071 (6)
O3	0.0296 (6)	0.0394 (7)	0.0263 (7)	0.0000 (5)	0.0050 (5)	0.0082 (6)

### Geometric parameters (Å, º)

**Table:**

N1—C11	1.5077 (19)	C12—C22	1.522 (2)
N1—C12	1.510 (2)	C12—C62	1.526 (2)
N1—H1N	0.91 (2)	C12—H12	1.0000
N1—H2N	0.86 (2)	C22—C32	1.527 (2)
C11—C21	1.521 (2)	C22—H22A	0.9900
C11—C61	1.524 (2)	C22—H22B	0.9900
C11—H11	1.0000	C32—C42	1.526 (2)
C21—C31	1.532 (2)	C32—H32A	0.9900
C21—H21A	0.9900	C32—H32B	0.9900
C21—H21B	0.9900	C42—C52	1.525 (2)
C31—C41	1.525 (2)	C42—H42A	0.9900
C31—H31A	0.9900	C42—H42B	0.9900
C31—H31B	0.9900	C52—C62	1.536 (2)
C41—C51	1.533 (3)	C52—H52A	0.9900
C41—H41A	0.9900	C52—H52B	0.9900
C41—H41B	0.9900	C62—H62A	0.9900
C51—C61	1.528 (2)	C62—H62B	0.9900
C51—H51A	0.9900	N2—O1	1.2353 (18)
C51—H51B	0.9900	N2—O3	1.255 (2)
C61—H61A	0.9900	N2—O2	1.258 (2)
C61—H61B	0.9900		
			
C11—N1—C12	117.34 (12)	H61A—C61—H61B	108.1
C11—N1—H1N	107.9 (13)	N1—C12—C22	107.92 (12)
C12—N1—H1N	109.3 (13)	N1—C12—C62	111.45 (12)
C11—N1—H2N	109.0 (14)	C22—C12—C62	111.51 (12)
C12—N1—H2N	105.4 (14)	N1—C12—H12	108.6
H1N—N1—H2N	108 (2)	C22—C12—H12	108.6
N1—C11—C21	110.62 (13)	C62—C12—H12	108.6
N1—C11—C61	108.83 (13)	C12—C22—C32	109.96 (13)
C21—C11—C61	111.20 (13)	C12—C22—H22A	109.7
N1—C11—H11	108.7	C32—C22—H22A	109.7
C21—C11—H11	108.7	C12—C22—H22B	109.7
C61—C11—H11	108.7	C32—C22—H22B	109.7
C11—C21—C31	110.42 (14)	H22A—C22—H22B	108.2
C11—C21—H21A	109.6	C42—C32—C22	110.87 (13)
C31—C21—H21A	109.6	C42—C32—H32A	109.5
C11—C21—H21B	109.6	C22—C32—H32A	109.5
C31—C21—H21B	109.6	C42—C32—H32B	109.5
H21A—C21—H21B	108.1	C22—C32—H32B	109.5
C41—C31—C21	111.49 (14)	H32A—C32—H32B	108.1
C41—C31—H31A	109.3	C52—C42—C32	111.30 (13)
C21—C31—H31A	109.3	C52—C42—H42A	109.4
C41—C31—H31B	109.3	C32—C42—H42A	109.4
C21—C31—H31B	109.3	C52—C42—H42B	109.4
H31A—C31—H31B	108.0	C32—C42—H42B	109.4
C31—C41—C51	110.99 (13)	H42A—C42—H42B	108.0
C31—C41—H41A	109.4	C42—C52—C62	111.46 (14)
C51—C41—H41A	109.4	C42—C52—H52A	109.3
C31—C41—H41B	109.4	C62—C52—H52A	109.3
C51—C41—H41B	109.4	C42—C52—H52B	109.3
H41A—C41—H41B	108.0	C62—C52—H52B	109.3
C61—C51—C41	110.81 (14)	H52A—C52—H52B	108.0
C61—C51—H51A	109.5	C12—C62—C52	109.50 (13)
C41—C51—H51A	109.5	C12—C62—H62A	109.8
C61—C51—H51B	109.5	C52—C62—H62A	109.8
C41—C51—H51B	109.5	C12—C62—H62B	109.8
H51A—C51—H51B	108.1	C52—C62—H62B	109.8
C11—C61—C51	110.18 (13)	H62A—C62—H62B	108.2
C11—C61—H61A	109.6	O1—N2—O3	121.19 (15)
C51—C61—H61A	109.6	O1—N2—O2	120.95 (16)
C11—C61—H61B	109.6	O3—N2—O2	117.86 (14)
C51—C61—H61B	109.6		
			
C12—N1—C11—C21	−56.99 (17)	C11—N1—C12—C22	−178.34 (13)
C12—N1—C11—C61	−179.43 (13)	C11—N1—C12—C62	−55.58 (18)
N1—C11—C21—C31	−178.14 (13)	N1—C12—C22—C32	−178.66 (12)
C61—C11—C21—C31	−57.10 (18)	C62—C12—C22—C32	58.62 (18)
C11—C21—C31—C41	55.47 (18)	C12—C22—C32—C42	−56.86 (18)
C21—C31—C41—C51	−55.0 (2)	C22—C32—C42—C52	55.7 (2)
C31—C41—C51—C61	55.81 (19)	C32—C42—C52—C62	−55.4 (2)
N1—C11—C61—C51	−179.74 (13)	N1—C12—C62—C52	−178.37 (13)
C21—C11—C61—C51	58.16 (18)	C22—C12—C62—C52	−57.70 (18)
C41—C51—C61—C11	−57.12 (18)	C42—C52—C62—C12	55.69 (19)

### Hydrogen-bond geometry (Å, º)

**Table:**

*D*—H···*A*	*D*—H	H···*A*	*D*···*A*	*D*—H···*A*
N1—H1*N*···O2	0.91 (2)	2.56 (2)	3.292 (2)	138.2 (17)
N1—H1*N*···O3	0.91 (2)	2.01 (2)	2.8988 (19)	166.7 (19)
N1—H2*N*···O2^i^	0.86 (2)	1.98 (2)	2.799 (2)	157.6 (19)
C11—H11···O1^ii^	1.00	2.45	3.347 (2)	149
C12—H12···O3^iii^	1.00	2.52	3.456 (3)	156
C22—H22*B*···O2	0.99	2.53	3.309 (2)	136
C62—H62*A*···O1^ii^	0.99	2.59	3.506 (2)	153

Symmetry codes: (i) −*x*+1, −*y*+1, *z*−1/2; (ii) *x*+1, *y*, *z*; (iii) −*x*+1, −*y*+1, *z*+1/2.
