# Supplementary figures and images for: Crystal structure of di­cyclo­hexyl­ammonium nitrate(V)
Source: Acta Crystallogr E Crystallogr Commun. 2015 Oct 24;71(Pt 11):o878–9. doi: 10.1107/S2056989015019386 (PMC4645052; doi:10.1107/S2056989015019386)

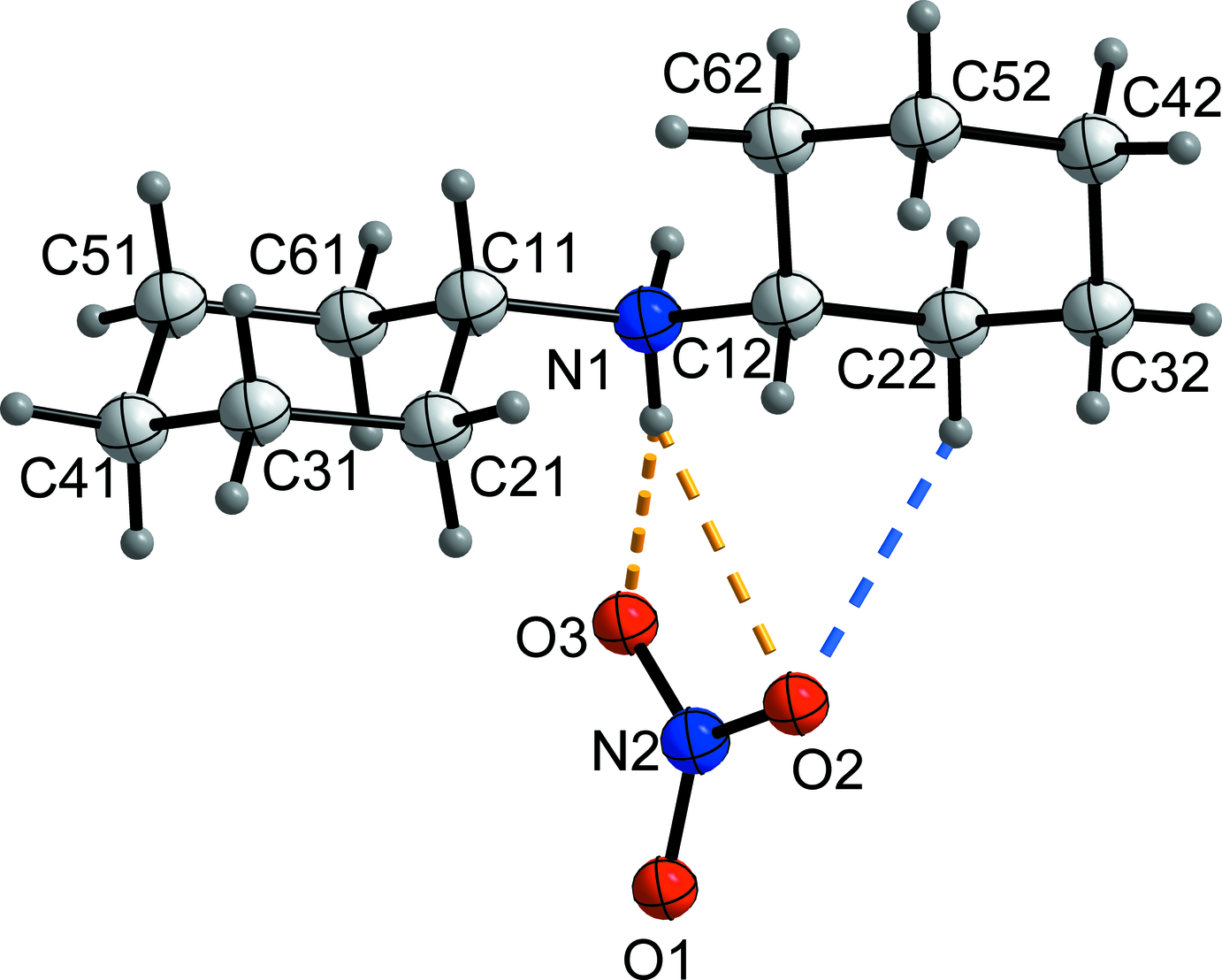

Supplement: Supplementary file 5 [file e-71-0o878-fig1.tif]

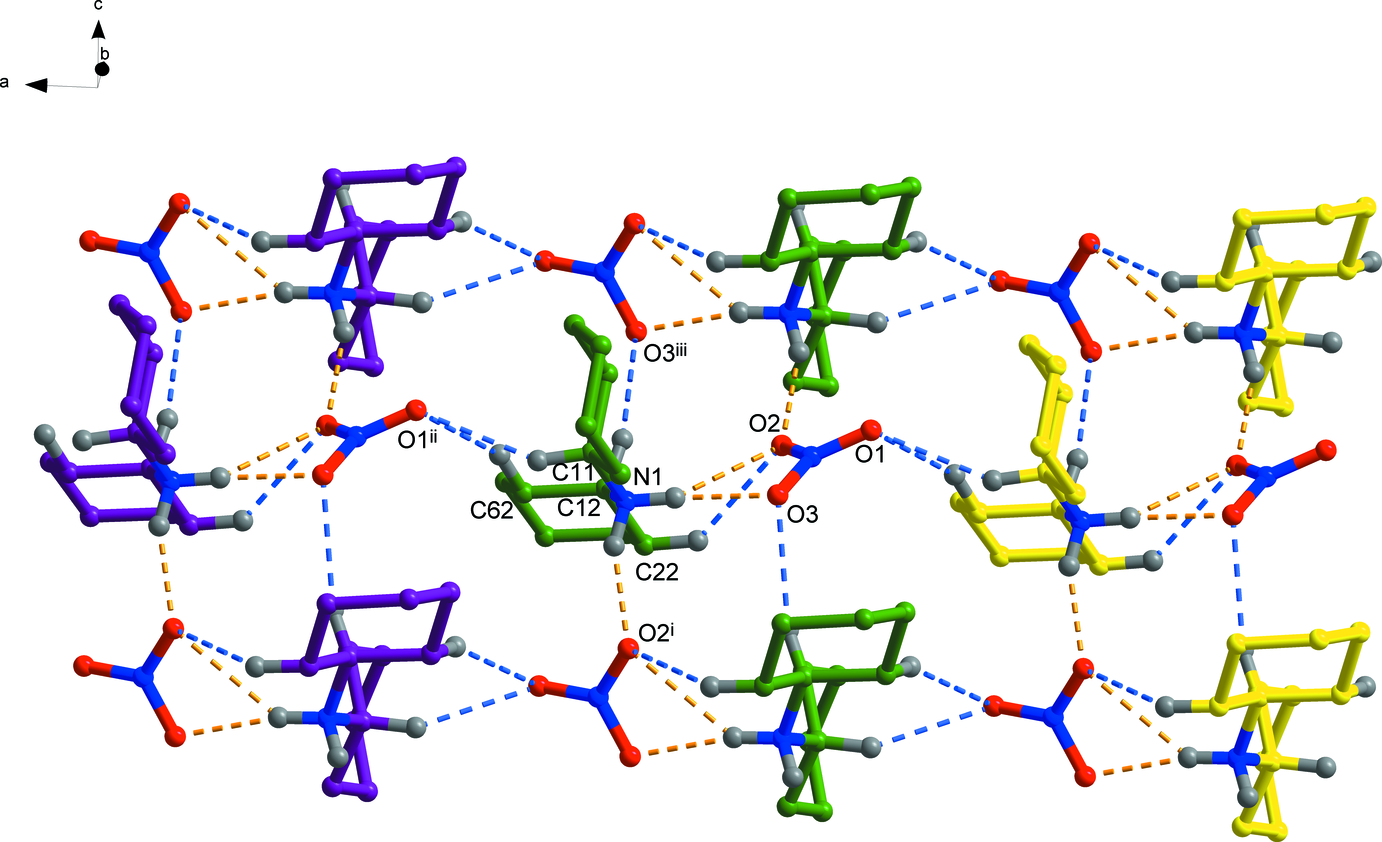

Supplement: Supplementary file 6 [file e-71-0o878-fig2.tif]
